# A case of acquired von Willebrand disease in severe pediatric pulmonary hypertension contributing to bleeding following reverse Potts shunt

**DOI:** 10.1002/pul2.12042

**Published:** 2022-02-04

**Authors:** Rachel T. Sullivan, Clara Lo, Elisabeth Martin, Rebecca J. Kameny, Rachel K. Hopper

**Affiliations:** ^1^ Department of Pediatrics, Division of Pediatric Cardiology, Stanford University School of Medicine Lucile Packard Children's Hospital Stanford Palo Alto California USA; ^2^ Department of Pediatrics, Division of Pediatric Hematology/Oncology, Stanford University School of Medicine Lucile Packard Children's Hospital Stanford Palo Alto California USA; ^3^ Department of Cardiothoracic Surgery, Division of Pediatric Cardiac Surgery, Stanford University School of Medicine Lucile Packard Children's Hospital Stanford Palo Alto California USA

**Keywords:** pediatric cardiovascular disease, pulmonary arterial hypertension, von Willebrand factor

## Abstract

The reverse Potts shunt is increasingly used as a palliative measure for end‐stage pulmonary arterial hypertension (PAH) as a means to offload the right ventricle and improve functional status. This case report describes a child who developed significant hemothorax after reverse Potts shunt that required surgical exploration, blood product administration, and prolonged intensive care hospitalization. Despite lack of preoperative bleeding symptoms, testing revealed acquired von Willebrand disease (aVWD), with subsequent resolution of bleeding. Alterations in von Willebrand factor, including aVWD, have been reported in children with severe PAH but have not previously been associated with bleeding after reverse Potts shunt procedure. As bleeding is a recognized postoperative morbidity in PAH patients undergoing reverse Potts shunt, we highlight a potential role for preoperative testing for aVWD as perioperative factor replacement therapy may improve postoperative outcomes.

## CASE DESCRIPTION

After presenting with syncope, a 10‐year‐old male was diagnosed with heritable pulmonary arterial hypertension (PAH) secondary to a maternally inherited pathogenic mutation in bone morphogenic protein receptor type 2. Cardiac catheterization at diagnosis demonstrated moderate PAH and preserved cardiac output with the following hemodynamic indices: mean pulmonary arterial pressure 46 mmHg, pulmonary artery wedge pressure 11 mmHg, cardiac index 3.8 L/min/m^2^, and indexed pulmonary vascular resistance 8.9 Wood units*m^2^. Acute vasoreactivity testing was negative. He was started on dual pulmonary vasodilator therapy with tadalafil and ambrisentan.

Despite initial mild symptomatic and echocardiographic improvement, he developed echocardiographic evidence of worsening (suprasystemic) pulmonary arterial pressure and right ventricular dysfunction approximately 9 months after diagnosis. Despite the escalation in therapy with the addition of subcutaneous treprostinil, the patient had two syncopal episodes with continued evidence of suprasystemic pulmonary artery pressures and worsening right ventricular function. Systemic hypotension and severe gastrointestinal symptoms limited increase in the dose of treprostinil above 27 ng/kg/min despite multiple attempts. Accordingly, the family opted to pursue surgical reverse Potts shunt. Lung transplant evaluation was undertaken before surgical reverse Potts shunt to allow for expedited listing in the event of clinical deterioration postoperatively.

Surgical reverse Potts shunt was performed using a 10 mm Hemashield graft via median sternotomy. Postoperative course was complicated by a spontaneous left hemothorax and pericardial effusion with prolonged sanguineous chest tube drainage, which persisted despite multiple transfusions of packed red blood cells, platelets, and fresh frozen plasma, as well as recombinant factor VIIa. On postoperative Day 6, the patient returned to the operating room for chest exploration for a source of the ongoing bleeding. No active bleeding was identified, and hemopericardium and hemothorax were evacuated. Hematologic testing was consistent with acquired von Willebrand disease (aVWD), suggested by decreased high molecular weight (HMW) von Willebrand factor (vWF) multimers and lack of family history of bleeding. Additional labs included discordant von Willebrand activity to antigen ratio (vWF activity 170% [reference range: 54%–137%]; von Willebrand antigen 235% [reference range: 56%–123%]; ratio: 0.73) and prolonged platelet function assay (>300 s; reference range: 94 s–193 s).

Hemothorax resolved without the need for vWF/factor VIII replacement therapy. vWF/factor VIII replacement was given empirically before both chest tube and pacer wire removal, with no postremoval bleeding. He was discharged 26 days postoperatively (21 days intensive care unit) on unchanged triple pulmonary vasodilator therapy. Since discharge, activity tolerance is improved with no further syncope. Right ventricular function has improved significantly, with mild dilation and dysfunction 8 months later. Notably, he has no presurgical history of easy bleeding or bruising, epistaxis, or gastrointestinal bleeding. He had one self‐limited episode of epistaxis since hospital discharge.

## DISCUSSION

This report describes a child with severe heritable PAH whose postoperative course following reverse Potts shunt was complicated by significant and prolonged hemothorax, likely exacerbated by unrecognized aVWD. The patient had no overt bleeding symptoms before surgery and no family history of bleeding disorders.

Von Willebrand disease is a bleeding disorder caused by abnormal quantity or function of vWF. Normally, vWF circulates as a HMW multimer and participates in platelet aggregation in response to vascular injury.^1^ The HMW vWF multimers are cleaved in a shear‐stress‐dependent manner by the metalloprotease enzyme ADAMTS13. While the more commonly encountered forms of vWD are inherited, aVWD is a result of abnormal degradation of vWF. In PAH, aVWD is thought to be secondary to shear stress‐related excessive cleavage of HMW vWF multimers.^2^ Patients with both idiopathic PAH and PAH secondary to congenital heart disease have been shown to have abnormal vWF antigen and activity levels as well as decreased HMW vWF multimers.^3^, ^4^ These laboratory abnormalities have demonstrated potential utility as a biomarker for disease severity, as high levels of vWF antigen are associated with poor short‐term and long‐term outcomes.^5^, ^6^ Cases of aVWD have been described in PAH, including in a pediatric cohort with a history of bleeding.^7^

Decreased or absent HMW vWF multimers is the gold standard for diagnosis of aVWD. Other laboratory abnormalities that can be observed include low vWF activity and antigen levels as well as a decreased ratio of vWF activity:antigen (typically <0.7). Notably, vWF activity and antigen values have low diagnostic sensitivity for aVWD (20%–26%) when each taken in isolation, and patients can have bleeding with decreased HMW vWF multimers as their only laboratory abnormality.^8^ While patients with aVWD typically have low vWF antigen levels, it can be elevated, as was the case in our patient, in aVWD secondary to cardiovascular disorders.^8^

The Potts shunt is a surgical systemic to left pulmonary arterial connection, which has been repurposed for the treatment of end‐stage pulmonary hypertension as a means to offload the right ventricle and improve functional status.^9^, ^10^ Bleeding is reported as a rare short‐term postoperative complication in the registry but may be more common based on anecdotal reports.^10^, ^11^ The frequency of significant bleeding is unknown given a relatively small number of reported cases and given that much of the current literature is focused largely on survival in this high‐risk population. Our patient's hospital course was significantly impacted due to prolonged bleeding, with need for an additional surgical procedure, longer intensive care and hospital length of stay, and significant blood product replacement requirements, the use of which may increase sensitization in this patient who may require future lung transplantation.

This is the first case report of significant postoperative bleeding related to aVWD after reverse Potts shunt. Preoperative knowledge of this diagnosis would likely have altered management, with empiric preprocedure vWF/factor VIII replacement therapy and additional replacement for any significant postoperative bleeding. Prior reports of vWF alteration in severe PAH suggests that this population may have a significant risk for subclinical aVWD that could be exposed at the time of the major surgical procedure. Therefore, we advocate for preoperative testing for aVWD in children undergoing reverse Potts shunt as specific therapy has the potential to diminish postoperative bleeding and improve patient outcomes.

## CONFLICT OF INTERESTS

The authors declare that there are no conflict of interests.

## AUTHOR CONTRIBUTIONS

Rachel T. Sullivan was responsible for the primary composition of the written manuscript draft. Clara Lo contributed to content regarding the hematologic diagnosis of acquired von Willebrand disease. Elisabeth Martin and Rebecca J. Kameny contributed to the discussion of surgical and cardiac critical care management. Rachel K. Hopper contributed to case selection, manuscript editing, and preparation. All authors were involved in the revision and final approval of this article.

## ETHICS STATEMENT

Ethical review was not required by the Institutional Review Board. Informed consent was obtained from the patient's father for the publication of patient information.
